# Epidemiology and Population Structure of Serotypes 1, 5 and 7F Carried by Children in Portugal from 1996-2010 before Introduction of the 10-Valent and 13-Valent Pneumococcal Conjugate Vaccines

**DOI:** 10.1371/journal.pone.0075442

**Published:** 2013-09-18

**Authors:** Sónia T. Almeida, Hermínia de Lencastre, Raquel Sá-Leão

**Affiliations:** 1 Laboratory of Molecular Microbiology of Human Pathogens, Universidade Nova de Lisboa, Oeiras, Portugal; 2 Laboratory of Molecular Genetics, Instituto de Tecnologia Química e Biológica, Universidade Nova de Lisboa, Oeiras, Portugal; 3 Laboratory of Microbiology, The Rockefeller University, New York, New York, United States of America; University of Malaya, Malaysia

## Abstract

Among the over 90 serotypes of *Streptococcus pneumoniae* described, serotypes 1, 5, and 7F account for a significant proportion of invasive disease worldwide and are now covered by the most recent 10- and 13-valent pneumococcal conjugate vaccines (PCVs). The epidemiology of these serotypes in carriage remains poorly studied because they are rarely detected. We aimed to gain insights into the epidemiology and population structure of serotypes 1, 5 and 7F carried by children in Portugal before PCV10 and PCV13 became widely used. Isolates obtained in cross-sectional studies carried out over a 15-year period (1996–2010) were retrospectively pooled and characterized. Of 5,123 pneumococci obtained, 70 were associated with serotypes 1 (n = 21), 5 (n = 7), and 7F (n = 42). The highest prevalence detected was 3.3% for serotype 1 in 2006, 1% for serotype 5 in 2009, and 3.3% for serotype 7F in 2006; Serotype 1 was associated with PMEN international clones Sweden^1^-28(ST306) and Sweden^1^-40(ST304); serotype 5 was associated with Colombia^5^-19(ST289); and serotype 7F was associated with Netherlands^7F^-39(ST191). All these isolates were fully susceptible. Most carriers of serotypes 1 (86%), 5 (86%), and 7F (91%) were older than two years but a significant association with older age was only observed for serotype 7F (p = 0.006). Evidence for cross-transmission was obtained. In conclusion, we were able to detect and characterize the rarely carried serotypes 1, 5, and 7F among healthy children in Portugal. These data will constitute an important baseline for upcoming surveillance studies aimed to establish the impact of novel PCVs targeting these serotypes in carriage.

## Introduction

*Streptococcus pneumoniae* (or pneumococcus) is not only a major human pathogen but also a commensal of the human nasopharynx. As a pathogen, *S. pneumoniae* is a frequent cause of otitis media, pneumonia, bacteremia and meningitis, especially among children under five years of age, the immunocompromised of all ages, and the elderly [1]. Among the over 90 capsular types described to date, their propensity to be carried and/or cause disease varies widely [2], [3].

In a review conducted by Hausdorff et al., serotypes 1, 5, and 7F accounted for a significant proportion of pneumococcal invasive disease particularly in Africa, Asia, and Latin America [4]. In addition, other studies have indicated that serotypes 1, 5 and 7F have a high invasive disease potential [2], [3], [5]. Serotypes 1 and 5 have been frequently associated with outbreaks, especially in crowded or closed communities [6], [7], [8], [9]. The few studies that have looked at the temporal distribution of serotypes over a large time period identified secular trends, which remain poorly understood [10], [11]. These serotypes are now included in the most recent pneumococcal conjugate vaccines, i.e., the 10-valent and 13-valent pneumococcal conjugate vaccines (PCV10 and PCV13, respectively).

During the past decade, an increase in serotypes 1 and 7F in invasive disease has been reported in some European countries such as Portugal, Spain, France, Belgium and UK [12], [13], [14], [15], [16]. In addition, serotype 5 was increasingly detected in Spain [13]. The few studies that looked at the genotype of these isolates found that most isolates were representatives or close relatives of PMEN clones typically associated with these serotypes, namely, Sweden^1^-28 (ST306), Sweden^1^-40 (ST304), Colombia^5^-19 (ST289) and Netherlands^7F^-39 (ST191) [12], [13], [14], [16], [17]. These serotypes and associated clones are known to be mostly antimicrobial susceptible.

Of interest, a recent genomic analysis of a highly virulent serotype 1 clone (ST217) in circulation in West Africa, found that the gene *comE*, an essential determinant of the competence operon (coding for the cognate response regulator) was partially deleted [18]. As horizontal gene transfer in pneumococci occurs typically during the competent state, the authors hypothesized that the limited genetic diversity of serotype 1, and the antimicrobial susceptible phenotype, could be linked to this observation. Other competent genes such as *comC* and c*omD*, coding for the competence stimulating peptide (CSP) and the histidine kinase sensor, respectively, were intact.

In contrast with cumulative findings regarding the role of serotypes 1, 5 and 7F in disease, less is known about its epidemiology in carriage. This may be attributed to the fact that these serotypes are rarely carried [5] hampering the chance of detecting them in point-prevalence studies. We have previously documented an apparent increase in asymptomatic carriage of serotypes 1 and 7F among healthy children attending day-care centers when the prevalence of these serotypes was compared between 2006, i.e., 5 years after the introduction of 7-valent pneumococcal conjugate vaccine (PCV7) in the Portuguese market, with 2001 (pre-vaccine era) [19], [20]. Recently, a report from Belgium described the isolation of serotype 1 from school children with carriage rates reaching up to 3% of the isolates [21]. For serotype 5 the data is much scarcer with only sporadic carriage being reported [22].

In Portugal, PCV7 became commercially available in June 2001, PCV10 in April 2009, and, in January 2010, PCV13 replaced PCV7. None of these vaccines has been introduced in the National Immunization Plan, nor is reimbursed by the state. Still, usage based on national sales data indicates PCVs were widely used. In particular, in 2009, when PCV10 became available, the usage of PCV10 and PCV7 among the target group has been estimated, based on sales data, to be 13% and 62%, respectively, with no evidence of a significant catch-up program (source: IMS and INE/National Statistics Institute) [12], [20].

This study aimed to describe the epidemiology and population structure of serotypes 1, 5 and 7F carried by children in Portugal before the introduction of PCV10 and PCV13. Since these serotypes are rarely carried, we retrospectively pooled all isolates obtained in point prevalence studies performed over a 15-year period (1996–2010). We show that serotypes 1, 5 and 7F can be detected in carriage, are able to transmit between children attending day-care centers, and are highly clonal. The information obtained will be essential to monitor the impact of PCV10 and PCV13 in carriage of these serotypes. As a secondary objective, we performed a genetic screening for the presence of essential competence genes.

## Methods

### Ethics statement

Approval for the sampling between 1996 and 2003 was obtained from the Regional Education Director from the Ministry of Education. Between 2006 and 2010, the study was registered at Health Care Centers of Oeiras and Montemor-o-Novo that report to Administração Regional de Saúde (ARS, “Regional Health Administration”) of Lisboa e Vale do Tejo, and Alentejo, respectively, from the Ministry of Health. Studies were approved by the Directors of Health Care Centers. All studies were approved by the directors of all day care centers. Signed informed consent was obtained from the parents or guardians of participating children. All samples were numerically coded upon sample collection and processed anonymously.

### Bacterial isolates

In this retrospective study, pneumococcal strains of serotypes 1, 5 and 7F isolated from nasopharyngeal samples obtained from healthy children attending day care centers in three areas of Portugal - Lisbon, Oeiras and Montemor-o-Novo - were characterized. The isolates were recovered between the winter months of January to March, in eleven sampling periods between 1996 and 2010. These point prevalence studies occurred yearly with the exception of 2000, 2004, 2005 and 2008. Previous reports from these studies were confined to drug-resistant isolates obtained between 1996 and 2007 [23], [24], [25].

Pneumococcal identification was performed as described before [25]. Isolates were serotyped by multiplex PCR as previously described [26] using the primers available at http://www.cdc.gov/ncidod/biotech/strep/pcr.htm and/or by the Quellung reaction with pneumococcal antisera commercially available (Statens Serum Institute, Copenhagen, Denmark).

### Antimicrobial susceptibility testing

Susceptibility to chloramphenicol, erythromycin, clindamycin, tetracycline and cotrimoxazole was tested by disk diffusion, according to the CLSI guidelines and interpretive criteria [27]. MICs to penicillin were determined by E-test according to the manufacturer’s instructions.

### Multilocus sequence typing

MLST was performed essentially as described using primers with universal M13 tails [28]. Sequencing reactions were performed at Macrogen (Amsterdam, The Netherlands). Sequences were analyzed with the Bionumerics software (Applied Maths, Gent, Belgium). Allele numbers and sequence types (ST) were determined using the website http://www.mlst.net for *Streptococcus pneumoniae*.

### Screening for competence determinants

Genetic changes in pneumococci occur by homologous recombination, where the competence state is induced by the presence of a competence-stimulating peptide (CSP) that is recognized by a two-component regulatory system, which includes the histidine kinase sensor (ComD) and its cognate response regulator (ComE) [29]. To investigate which allele of CSP was associated with the isolates under study, a duplex PCR screening for CSP1 and CSP2 was used as previously described [30]. Detection of *comD* and *comE* was performed by PCR. Primers were designed based on the TIGR4 sequence available at GenBank (accession no. NC_003028.3). Primers’ sequences were: *comD-*fwd 5′-ATTAAAGGTGGGGAGATGAGG-3′ and *comD-*rev 5′-CCAGCATAATCATGTCG-3′ (fragment length of 841bp) for *comD* gene; and *comE*-fwd 5′-ACGGACCTTCTATCTGTAGC-3′ and *comE*-rev 5′-ACTAAGGGAAGAAATCGCGG-3′ (fragment length of 945bp) for *comE* gene. To test possible cross-reactivity of these primers, BLAST (Basic Local Alignment Search Tool) was used.

### Statistical analysis

In order to explore a possible association between carriage of a specific serotype and age, statistical analysis was performed using the χ^2^ test. A p-value of <0.05 was considered statistically significant.

## Results

### Carriage of serotypes 1, 5, and 7F

**Figure 1:**
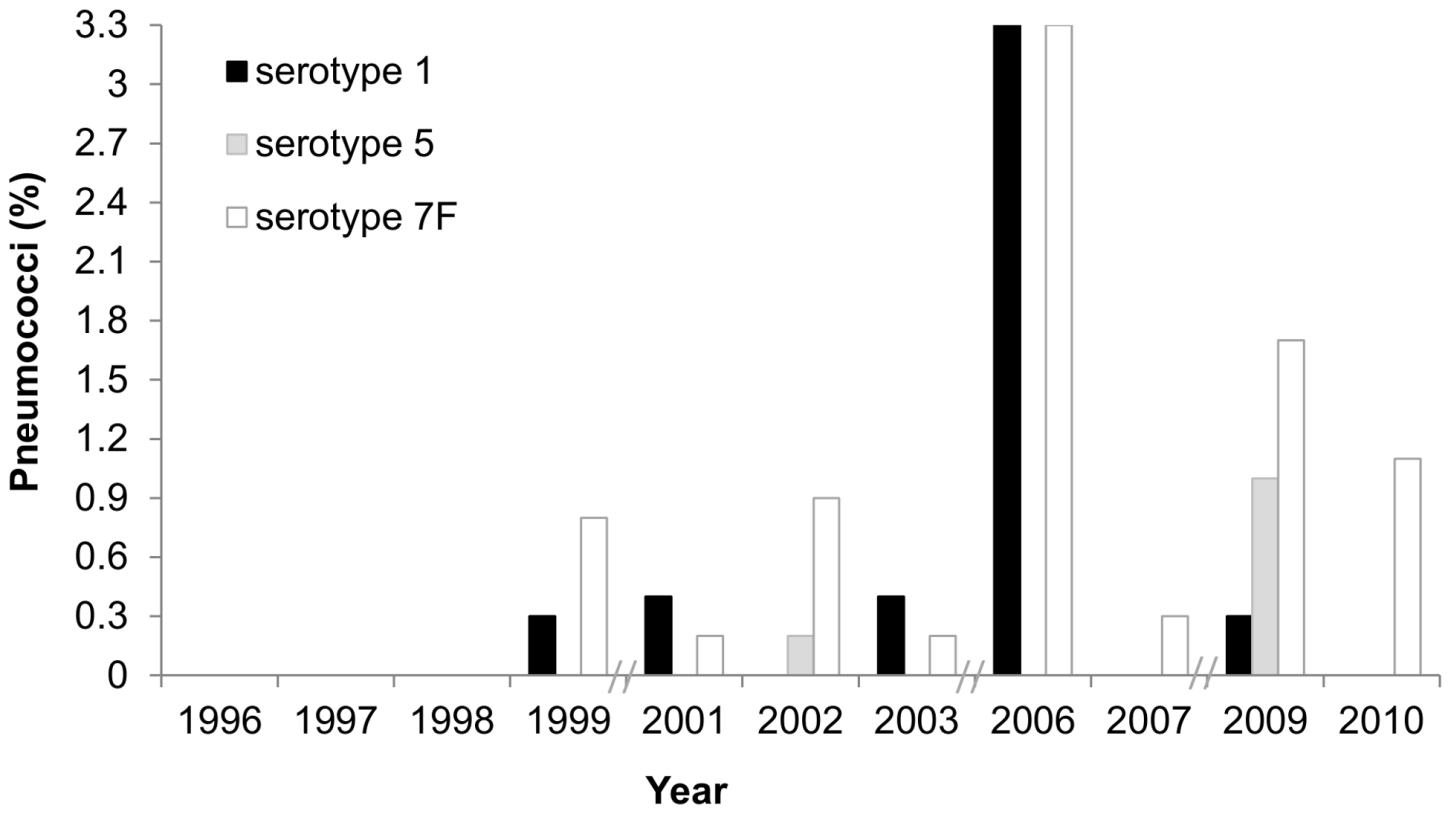
Prevalence of serotypes 1, 5, and 7F over time.

Between 1996 and 2010, 8,330 nasopharyngeal samples were obtained. Overall, 61.5% (5,123) had pneumococci. The number of isolates associated with serotypes 1, 5, and 7F was 21 (0.41%), 7 (0.14%), and 42 (0.82%), respectively. These serotypes were rarely isolated as shown in Figure 1. The highest prevalence detected for each of them was 3.3% for serotype 1 in 2006, 1% for serotype 5 in 2009, and 3.3% for serotype 7F in 2006.

### Characteristics of serotypes 1, 5, and 7F carried isolates

All isolates (n = 70) were susceptible to the six antimicrobial agents tested. Molecular typing by MLST identified three STs associated with serotype 1: ST306 (n = 16), ST228 (DLV of ST306, n = 3), and ST304 (TLV of ST306, n = 2). All these isolates belong to the clonal complex CC306 and are representatives of the PMEN international clones Sweden^1^-28 (ST306) and Sweden^1^-40 (ST304). The seven serotype 5 isolates were associated with ST1223, a SLV of the PMEN Colombia^5^-19 (ST289) clone. Among the serotype 7F isolates, all but one isolate were associated with ST191, the PMEN clone Netherlands^7F^-39 (ST191). The other 7F isolate was associated with ST4771, a SLV of ST191.

### Epidemiology of carriage

**Figure 2:**
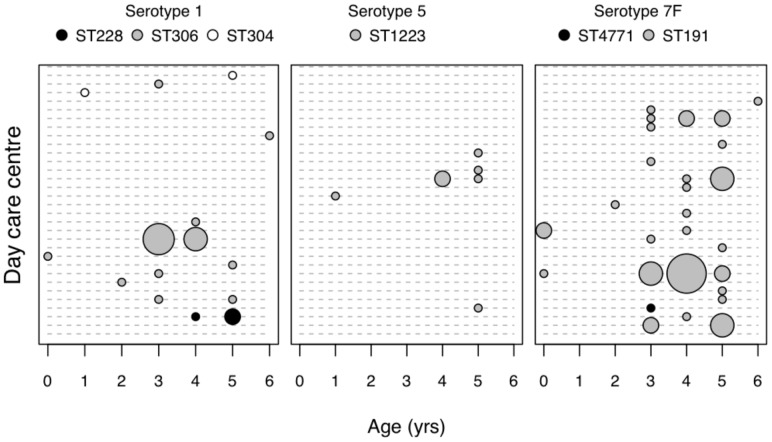
Distribution of serotypes 1, 5, and 7F isolates according to age and day-care center. In the yy axis, each row corresponds to a unique association of day care center unit and sampling year. The size of the circles is proportional to the number of isolates found and ranges between 1 and 5. Lineages, as determined by MLST, are indicated.

Figure 2 shows the distribution of serotypes 1, 5, and 7F according to age of the carriers and day care center. Analysis of Figure 2 suggested that there might be an association between carriage and age as most carriers of serotypes 1 (86%), 5 (86%), and 7F (91%) were older than two years. This was explored using the χ^2^ statistic taking into account that, overall, 1,451 children were aged 0–2 years old, and 3,672 were aged 3–6 years old. Non-significant associations were found for serotypes 1 (χ^2^ = 2.047, p = 0.15) and 5 (χ^2^ = 0.68, p = 0.41). A significant association of older age and carriage of serotype 7F was observed (χ^2^ = 7.372, p = 0.006).

Although these serotypes were detected sporadically in each unit, there was evidence of cross-transmission for serotypes 1 and 7F in some units (Figure 2). For example, 33% of the isolates associated with serotype 1 were recovered from a single unit from children aged 3–4 years old; similarly, 26% of the isolates associated with serotype 7F were recovered from a single unit from children aged 0, 3, 4, and 5 years old.

### Screening for competence determinants

All isolates were of pherotype CSP1. Screening of the presence of *comD* by PCR, yielded a fragment of the expected size in all strains. Likewise, screening for the presence of *comE* by PCR, yielded a fragment of the expected size in all but four strains. The latter were all of serotype 1 (three were of ST306, one was of ST304) and the amplicon had an estimated size of 1.5–2 kb instead of 945 bp. Still, sequencing of the amplicon revealed the presence of an intact *comE* (Genbank accession number KF523383).

## Discussion

The aim of this work was to describe the epidemiology and the population structure of serotypes 1, 5, and 7F carried by children in Portugal in the years preceding the introduction of PCV10 and PCV13, the first pneumococcal conjugate vaccines targeting these serotypes that became commercially available.

A strength of our study was the fact that we had access to pre-PCV10/PCV13 carriage isolates recovered during eleven years encompassing 1996–2010. Although these serotypes are frequently not detected in carriage studies, we were able to identify representatives of all three serotypes when large collections of carried isolates were retrospectively analyzed. Overall, we confirmed they are rarely carried, each contributing to less than 1% of the entire pneumococcal population under study. Still, in 2006 serotypes 1 and 7F reached 3.3% of all isolates and were cross-transmitted within day-care centers, in line with evidence that these serotypes have a high disease potential and an epidemic nature [2], [3], [5]. Of interest, in recent years the proportion of pneumococcal disease caused by serotypes 1, and 7F has increased in several European countries such as Spain, France, Belgium and England and Wales [15]. In addition, an increase in serotype 5 has also been noted [13]. In particular, in Portugal, by 2008, serotypes 1 and 7F were the first and third, respectively, most frequently serotypes found among pediatric invasive disease, reaching altogether 36% of all isolates [12]. Although it may be tempting to associate the increase of these serotypes to the introduction of PCV7 other scenarios cannot be ruled out. In particular, secular trends have been observed and cyclical outbreaks of serotypes 1 and 5 have been described [6], [7], [8], [9], [10], [11].

In this study, most isolates of serotypes 1, 5, and 7F were isolated from carriers older than 2 years of age although, in contrast with studies looking at disease isolates, a statistically significant association with age was only detected for serotype 7F [5], [12]. It is probable that our study was underpowered to detect an association between age and carriage of serotypes 1 and 5. In fact, based on the age distribution of our study and the prevalence of serotypes 1 and 5 in each age group, a population size of over 10,000 and 30,000 individuals, respectively, would have been needed to achieve significant differences.

By MLST, the three serotypes were highly clonal and all were representatives or close relatives of PMEN international clones previously described (Sweden^1^-28 (ST306), Sweden^1^-40 (ST304), Colombia^5^-19 (ST289) and Netherlands^7F^-39 (ST191)). A literature review of lineages associated with disease caused by these serotypes in Portugal and other European countries in recent years showed, maybe not surprisingly, that the same lineages had been found in disease. In particular, Sweden^1^-28 (ST306), and Sweden^1^-40 (ST304) have been associated with pneumonia and invasive disease in several countries such as Portugal, Spain, Belgium, and UK [13], [14], [16], [17], [21]. Lineage Colombia^5^-19, was isolated from patients with IPD in Portugal, Spain, and UK [13], [14], [16]. Finally, Netherlands^7F^-39 has been associated with IPD in Portugal, Spain, and England and Wales [12], [13], [14], [16].

In our study, all isolates were fully susceptible to the antimicrobial agents tested. Other representatives of the same serotype/lineages reported in the abovementioned studies had, for the great majority, the same susceptibility pattern. The reasons for this are unclear but consistent worldwide and may be multifactorial. The fact that these serotypes are rarely carried suggests their exposure to antibiotics is infrequent resulting in low selective pressure. In addition, as carriage is essential for horizontal gene transfer, the opportunities for acquisition of resistance determinants, are probably rare. We explored whether the competence genes - *comC*, *comD* and *comE* - were present in the isolates under study, as this operon is essential for acquisition of exogenous DNA through genetic transformation. In fact, genomic analysis of the hypervirulent serotype 1 clone (ST217) circulating in Africa, has found that it lacks an intact response regulator *comE* essential for competence [18]. However, in our study, this was not the case, as all isolates seemed to carry *comC*, *comD* and *comE*. In addition, they were all associated with pherotype CSP1, the most common pherotype in this species [30]. Still, as no further characterization of the competence locus was done, definitive conclusions regarding its functionality could not be achieved.

In conclusion, in this study we were able to detect the rarely carried serotypes 1, 5, and 7F among healthy young carriers in Portugal. These serotypes seem to have an “outbreak-like” distribution being on one hand, rarely detected and, on the other, detected simultaneously from children sharing the same space. The lineages associated with these serotypes are the same found in disease in Portugal during the same years. As pneumococcal conjugate vaccines with expanded valency (PCV10 and PCV13) including these serotypes become available, it will be of interest to study their impact not only in disease, but also in carriage. In that sense, the data reported in this study, encompassing large carriage collections obtained over a 15-year period, will constitute an important baseline for upcoming surveillance initiatives.
